# Inherited Disease

**Published:** 1867-11

**Authors:** Henry S. Chase


### INHERITED DISEASES.

### A CASE—BY HENRY S. CHASE, D. D. S.

A CASE illustrating the above, came to my notice a few days since, so beautiful in its development that I must report it:

Mrs. D., German. Has right upper lateral incisor decayed, which I have plugged. She says her mother has the same tooth plugged, while all the others are sound. Two sisters have lost the same teeth by decay, while the others are good.

How important that this law of reproduction should be better understood! "Like produces like." If both parents before producing a living being like themselves, would have their own teeth put into a healthy condition, is it too much to affirm that the offspring would have a better denture than if conception occurred under opposite circumstances?

This is a *fact* in Dental hygiene which cannot be disproved.

Not only are *natural* habits and forms of body transmitted to offspring, but acquired forms also, in some cases. Thus have children not unfrequently wanted a member of the body which had by accident or disease been removed from the parent.

---

Cases analagous to the above come under the observation of nearly all experienced Dentists; but that does not render this case uninteresting. We have yet much to learn in regard to hereditary influence. That an artificial condition is transmissible from parent to offspring is denied by many who admit, as they must, that natural ones are. All will agree that tubercular disease is hereditary, yet that is an artificial condition. But the objector will say that the blood, the entire vital fluid, is contaminated, and that the case is not analagous to that of a lost organ, with the remaining organs of the system healthy. But instances are not uncommon like those alluded to in the closing lines of the above article. A noted one in comparative physiology was observed by us in our boyhood: A dog, the only one of the kind in the neighborhood, had his tail cut off when he was but a few weeks old. In a series of years he was repeatedly mated with a long-tailed bitch, other dogs being carefully excluded. In almost every litter short-tailed pups were found, whose form, color, and texture of hair, would have clearly shown their paternity had there been any room for doubt.

The advice to parents how to proceed "before producing a living being like themselves" is good; and even if they are not going to do any thing so marvelous, it would be well "to have their own teeth put into a healthy condition." W.

